# Estimation of Carbon Metabolism in *Saccharomyces cerevisiae* Acclimatized to Glycerol Assimilation with Quantitative PCR

**DOI:** 10.3390/microorganisms10061173

**Published:** 2022-06-07

**Authors:** Akihito Nakanishi, Kuan Zhang, Riri Matsumoto, Naotaka Yamamoto

**Affiliations:** 1 Graduate School of Bionics, Tokyo University of Technology, 1404-1 Katakuramachi, Hachioji 192-0982, Tokyo, Japan; d11210034f@edu.teu.ac.jp (K.Z.); g11220402a@edu.teu.ac.jp (N.Y.); 2 School of Bioscience and Biotechnology, Tokyo University of Technology, 1404-1 Katakuramachi, Hachioji 192-0982, Tokyo, Japan; b011929061@edu.teu.ac.jp

**Keywords:** *Saccharomyces cerevisiae*, glycerol acclimatization, carbon metabolism, transcriptomics

## Abstract

*Saccharomyces cerevisiae* has the potential to produce value-added chemicals; however, this strain is restricted by using glycerol as a carbon source. Although acclimatization of *S. cerevisiae* as a glycerol-assimilating strain was confirmed so far, the reason why *S. cerevisiae* can be acclimatized was not clear in detail with limited information on the metabolic changes. In this report, glycerol-assimilating strains from *S. cerevisiae* BY4741 were isolated, and the biomass production, ethanol fermentation, and transcription levels related to glycolysis and the tricarboxylic acid cycle under aerobic and slightly anaerobic conditions were analyzed. As the results show, although *µmax* was equal to 0.15 h^−1^ between wildtype and glycerol-assimilating strains in an aerobic culture including glucose, the differences in max biomass production and percentage yields of ethanol and transcription levels between the two strains were shown. In slightly anaerobic culture, the differences in transcription levels downstream of glycolysis were also displayed. In the case of the glycerol-assimilating strain with glycerol under aerobic conditions, although the transcription levels related to ethanol production were sufficient, the ethanol production was not detected. Additionally, the biomass production reached a plateau even in the culture containing sufficient glycerol, indicating that the redox imbalance even in the cells of the glycerol-acclimatized strain could disturb the utilization of glycerol. The obtained knowledge will promote the use of glycerol resources with the glycerol-acclimatized *S. cerevisiae* in view of carbon recycling.

## 1. Introduction

In industry, glycerol is abundantly produced as a byproduct. As an example, under biodiesel production, a considerable amount of crude glycerol is produced as a byproduct with its weight equivalent to 10% (*w*/*w*) of the produced biodiesel weight during transesterification with animal fats/vegetable oils and alcohols [1]. By producing not only biodiesel but also foodstuff [2], etc., a large amount of crude glycerol is produced and the price of the crude glycerol is discounted to around 225~235 USD ton^−1^ [3]; therefore, glycerol can be an attractive carbon resource. Additionally, the use of the crude glycerol can also be attractive because it deeply relates to carbon recycling societies. Therefore, research and development for the effective use of the promising glycerol are strongly required based on the abundance and carbon recycling [4]. Microbial utilization is one such attractive method since several microorganisms exhibit a tolerance to the contaminants in crude glycerol and an ability to utilize glycerol as an alternate carbon source for sugars [2]. So far, the method of using microorganisms has been evaluated directly to convert glycerol to the value-added chemicals [2]. In recent decades, *Saccharomyces cerevisiae* has attracted a lot of attention in the field of industrial biotechnology to produce biofuel, fine pharmaceutical chemicals, and many value-added substances [5] because the strain exhibits robustness towards several conditions in industrial bioprocesses [6], safety [7], low sensitivity to several stresses derived from methanol and heavy-ions unlike bacteria [8,9], abundantly available biological and genetic information [6,10], and easy manipulability for creating mutant strains [10]. However, *S. cerevisiae* shows higher utilization activity for glucose and much lower for glycerol, and the strain requires a long acclimatization period to utilize glycerol [11]. So far, unfortunately, information with regard to *S. cerevisiae* including the switching of intracellular metabolism by glycerol acclimatization has not been revealed in detail [12].

This study tried to evaluate the cell response of the glycerol-acclimatized strain derived from *S. cerevisiae*. A haploid strain *S. cerevisiae* BY4741 as the wildtype was cultivated, step-wise, with the restricted liquid media containing glycerol as a carbon source, and the isolates were obtained as glycerol-acclimatized strains derived from BY4741 on an agarose plate of synthetic glycerol medium. Afterwards, the acclimatized strains were aerobically cultivated in the synthetic media containing glucose or glycerol, respectively, and the different utilization activities were evaluated among the acclimatized strains and the wildtype. Additionally, the intracellular metabolism under aerobic and slightly anaerobic conditions (hereafter, simply anaerobic conditions) were determined using analyses of gene transcription levels with quantitative PCR (qPCR). Additionally, the transformation of the acclimatized strains was performed with the plasmid harboring the *S. cerevisiae ori* region, aiming to show the possibility of glycerol use with the transformant derived from the acclimatized strains. This study aims to disclose the response of the BY4741 wildtype strain under the glycerol condition. The obtained knowledge could estimate the metabolic reaction-limiting steps and be useful for creating glycerol-assimilating strains with genetic engineering for value-added products by using glycerol (e.g., waste glycerol as a by-product).

## 2. Materials and Methods

### 2.1. Experimentally Original Strains and Culturing Media

The *Saccharomyces cerevisiae* BY4741 strain (*MAT***a**, *his3Δ1*, *leu2Δ0*, *met15Δ0*, *ura3Δ0*) [13] was purchased from Funakoshi (Tokyo, Japan) as a laboratory haploid strain. Optical density at 600 nm (OD_600_) was measured to quantify the strain amount with U-2900 (Hitachi High-Technologies Corporation, Tokyo, Japan). The media used in this paper were used as mentioned below. Firstly, yeast extract peptone dextrose medium (YPD: 10 g·L^−1^ yeast extract, 20 g·L^−1^ peptone, 20 g·L^−1^ glucose), which was a nutrient-rich medium for *S. cerevisiae*, was used to stimulate *S. cerevisiae* from the glycerol stock. Secondly, yeast extract peptone glycerol medium (YPG: 10 g·L^−1^ yeast extract, 20 g·L^−1^ peptone, 20 g·L^−1^ glycerol), which changed only one of the YPD components from sugar glucose to sugar alcohol glycerol, was used to apply *S. cerevisiae* nutrient restriction. Thirdly, yeast nitrogen base glycerol medium (YNBG: 6.7 g·L^−1^ yeast nitrogen base without amino acids, 20 g·L^−1^ glycerol, 20 mg·L^−1^ L-histidine, 100 mg·L^−1^ L-leucine, 20 mg·L^−1^ L-methionine, 20 mg·L^−1^ uracil), which switched the nitrogen source based on YPG and synthetic defined medium as mentioned below, was used to pick up the glycerol-acclimatized strains. The medium could provide *S. cerevisiae* glycerol as the main carbon source. The YNBG plate contained 1.5% (*w*/*v*) agarose in YNBG to separate colonies. Fourthly, synthetic-defined medium (SD: 6.7 g·L^−1^ yeast nitrogen base without amino acids, 20 g·L^−1^ glucose, 20 mg·L^−1^ L-histidine, 100 mg·L^−1^ L-leucine, 20 mg·L^−1^ L-methionine, 20 mg·L^−1^ uracil), which contained glucose instead of glycerol in YNBG, was used to check *S. cerevisiae*’s biological properties [14,15].

### 2.2. Selection of Acclimatized Strains in YNBG Medium

**Figure 1:**
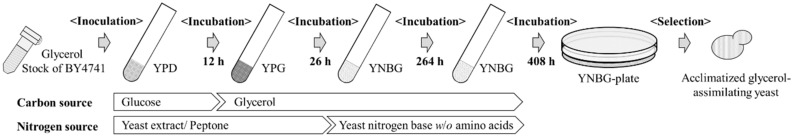
Overview of picking up glycerol acclimatized strains. BY4741 strains were acclimatized to glycerol assimilation gradually by nutrient pressure.

BY4741 wildtype strain was continuously cultured to be acclimatized as a glycerol-assimilating strain in restricted media of carbon sources under aerobic conditions (Figure 1). Stocked BY4741 was recovered in YPD medium at 140 spm (stroke per minutes) at 30 °C for 12 h. The recovered strain was cultured in YPG medium inoculated at initial OD_600_ = 0.1 at 140 spm at 30 °C for 26 h. Then, the strain was bred inoculated at the initial OD_600_ = 0.1 at 140 spm at 30 °C for 264 and 408 h in the YNBG media. Later, the diluted broth was spread on the YNBG plate, and the acclimatized glycerol-assimilating strains were isolated from a single colony.

### 2.3. Primary Evaluation of Growth of Isolated Glycerol-Acclimatized Strains in YNBG Medium

Twenty-one strains isolated from the YNBG plate were cultured aerobically in YNBG at 140 spm at 30 °C for 572 h. The pre-incubated strains were inoculated at initial OD_600_ = 0.01, and cultivated aerobically in YNBG medium at 140 spm at 30 °C for 503 h. The growth was evaluated with the values of OD_600_.

### 2.4. Secondary Evaluation of Growth of No.7 in YNBG Medium

The isolated strain no.7 was cultured aerobically in YNBG at 140 spm at 30 °C as a pre-cultivation. The pre-incubated no.7 at the middle of the exponential phase was harvested and inoculated as at initial OD_600_ = 0.01. The cultivation was performed in YNBG medium at 140 spm at 30 °C. Test-tubes were equipped with a silico plug under aerobic conditions and with a tightly held lid for anaerobic conditions.

### 2.5. Evaluation of Relative Growth Rate and Doubling Time

Relative growth rate (*µ*) and doubling time (*td*) were calculated using the formulas presented below (*t*_1_: sampling time 1 (*h*); *t*_2_: sampling time 2 (*h*) (*t*_2_ > *t*_1_); *S*_1_: scale of concentration indicated by OD_600_ at *t*_1_; *S*_2_: scale of concentration indicated by OD_600_ at *t*_2_).

$$\mu \;=\;\frac{ln(S_{2}/S_{1})}{\left(t_{2}\;-\;t_{1}\right)}\;\left(h^{-1}\right)$$

$$td\;=\;\frac{ln2}{\mu max}\;\left(h\right)$$

### 2.6. Determination of Biomass Production

Dry cell weights (DCW) of wildtype strain and no.7 were determined with calibration curves of OD_600_ versus DCW specifically to both strains, respectively. The biomass production was calculated with the DCW and its volume and the unit was defined as g·L^−1^.

### 2.7. Quantification of Carbon Source and Ethanol by HPLC

Under aerobic condition performed with test-tubes equipped with a silico plug, the cultivation of the wildtype strain was started at OD_600_ = 0.1 in SD, and no.7 strain was also started at OD_600_ = 0.1 in SD and YNBG at 140 spm at 30 °C, respectively. In total, 1 mL of each broth was collected at the middle of the exponential phase, and the supernatant was obtained by centrifugation at 5000× *g* for 1 min. On the other hand, under anaerobic conditions performed with test-tubes equipped with a tightly held lid, the wildtype strain was inoculated at OD_600_ = 1 in SD, and no.7 strain was also inoculated at OD_600_ = 1 in SD and YNBG at 140 spm at 30 °C, respectively. The anaerobic condition was set up by tightly closing the lid of the test-tube. Then, 1 mL of each broth was collected at 24 h, and the supernatant was obtained by centrifugation at 5000× *g* for 1 min. The supernatant was filtered with a Millex^®^ syringe filter (pore size 0.45 µm) (Merck Millipore Ltd., County Cork, Ireland), and the flow-through was analyzed with the HPLC system. The HPLC system consisted of a LC-20AD pump (Shimadzu, Kyoto, Japan), a CTO-10A column oven (Shimadzu), an SPD-20AV detector (Shimadzu), a Rezex^TM^ RHM-Monosaccharide H^+^ column (300 mm × 7.8 mm) (Phenomenex, Tokyo, Japan), and a 7725 injector (Rheodyne, Bensheim, Germany). The concentrations of glucose, glycerol, and ethanol were identified and quantified at 190 nm with chromatographic data monitored by the SPD-20AV detector. The mobile phase was water, the column oven was set at 30 °C, and the flow rate was 0.6 mL·min^−1^.

### 2.8. Confirmation of Relevance of Glycerol-Acclimatized Strain No.7 to Wildtype

Glycerol-acclimatized strain no.7 was determined as the strain derived from BY4741 by sequencing *glycerol 3-phosphatase 2* (*GPP2*). The gene of *GPP2* was amplified by PCR with the specific primer-pair for *GPP* (forward primer: atgggattgactactaaacctctatc; reverse primer: ccatttcaacagatcgtcc). The amplicon was sequenced by Macrogen Japan Corporation (Tokyo, Japan). Gene sequence information of BY4741 was obtained from the Basic Local Alignment Search Tool supported by the National Center for Biotechnology Information in the US.

### 2.9. Transcript Analysis

Wildtype and no.7 strains were cultivated in SD at 140 spm at 30 °C under aerobic/anaerobic conditions; no.7 strain was also in YNBG at 140 spm at 30 °C under aerobic/anaerobic conditions. Aerobic and anaerobic conditions were created with a test-tube equipped with a silico plug and a tightly held lid. The strains collected were approximately 5 mg estimated using an OD_600_-dry cell weight (DCW) calibration curve by centrifugation at 5000× *g* for 1 min. The collected strains were mixed with 50 µL of QIAzol Lysis Reagent (QIAGEN, Tokyo, Japan) and shaken for 5 min. After keeping the shaken samples at 23 °C for 5 min, 10 µL of chloroform was added and placed on ice for 3 min. The treated samples were centrifuged at 21,500× *g* for 1 min, and the supernatant was mixed with 25 µL of isopropanol. The supernatant was discarded after centrifugation at 21,500× *g* for 1 min and the precipitant was rinsed with 75% ethanol. The dried precipitant was dissolved in 10 µL of RNase free water. The prepared sample as total RNA was used to synthesize complementary DNA (cDNA) using a ReverTra Ace^®^ qPCR RT Master Mix with gDNA Remover (TOYOBO, Osaka, Japan). With the cDNA, qPCR was performed with THUNDERBIRD SYBR qPCR Mix (TOYOBO) using Mx qPCR Systems (Agilent, CA, USA). The average threshold cycle values were evaluated throughout the logarithmic amplification phase, and were normalized using the level of the *RDN18*. The qPCR primers (Table S1) were designed by Primer3Plus algorithm (https://dev.primer3plus.com/index.html (accessed on 6 January 2022)) based on each predicted gene sequence from the genome information. The abbreviated gene information is listed in Supplementary Table S1 and the primer information is also displayed in Supplementary Table S2.

### 2.10. Yeast Transformation

Transformation of glycerol-acclimatized no.7 was performed with pATP425 [16] using the lithium acetate method with a *S. cerevisiae* Direct Transformation kit (FUJIFILM Wako chemicals, Saitama, Japan). The transformants were isolated on a selective SD plate containing 1.5% (*w*/*v*) agarose at 30 °C for 2–3 days.

## 3. Results and Discussion

### 3.1. Overview of Picking up BY4741 Strains Acclimatized to Glycerol Assimilation

Yeast model strain *S. cerevisiae* BY4741 (wildtype strain, hereafter expressed simply ‘wildtype’) was pressured, stepwise, to be acclimatized for assimilating glycerol as the carbon source with the three media, YPD, YPG, and YNBG (Figure 1). By cultivating in YNBG, although the strains did not show increasing OD_600_ as cell-growth in the first cultivation for 264 h, the strains exhibited gradually enhancing OD_600_ to over 0.5 in the second cultivation for 408 h.

The glycerol utilization of *S. cerevisiae* was surveyed, and the findings support selection of the specific strains. The acclimatization of *S. cerevisiae* with a long cultivation time in the synthetic glycerol medium was also shown by previous reports [17]. The selection method with the stepwise carbon-restricted media was appropriate for isolating the glycerol-acclimatized strains in our study (Figure 1).

### 3.2. Growth Profiles of Glycerol-Acclimatized Strains

**Figure 2:**
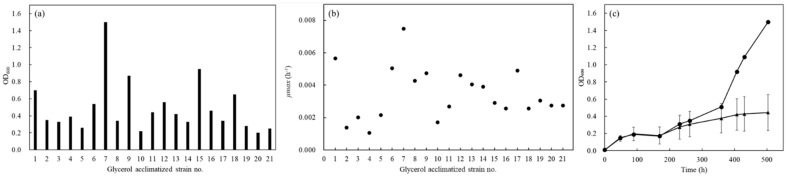
Growth profile of glycerol-acclimatized strains on YNBG medium after selection from the YNBG plate. (**a**) Growth of glycerol-acclimatized strains was evaluated with the OD_600_ value after 503 h cultivation in YNBG medium; (**b**) *µmax* of each glycerol-acclimatized strain in YNBG medium was displayed; (**c**) growth profiles of glycerol-acclimatized strains were evaluated using each OD_600_ value. The growth profiles are shown as symbols of circles (no.7) and triangles (averaged growth of glycerol-acclimatized strains without no.7). Error bars indicate standard deviation of OD_600_ of glycerol-acclimatized strains without no.7. The growth profiles in YNBG were used for the primary evaluation.

The cultivated group was acclimatized to utilize glycerol in the second YNBG cultivation. Finally, 21 strains were successfully picked up as the strains acclimatizing to utilize glycerol on the YNBG plate and named as no.1 to no.21. The growth properties of no.1~21 strains were analyzed primarily to select the most acclimatized strain in the group (Figure 2). In the group, the values of OD_600_ indicating growth widely ranged from 0.2 to 1.5 after 503 h of cultivation, and the OD-value of no.7 was a top score (Figure 2a). Max relative growth rates (*µmax*) of those segregants were 0.0011~0.0075 h^−1^ (Figure 2b), and the best scores of *µmax* and doubling time of no.7 were 0.0075 h^−1^ and 93 h. However, the lag phase of no.7 was over 169 h and there were few differences in the group. Strain no.7 showed different growth in the acclimatized group, especially after cultivation for 169 h, and the OD-value was 1.5-fold higher than the average of the values in the group (Figure 2c). The no.7 was evaluated to reveal the strain origin by comparing the DNA sequence of *GPP2*, and 753 bp of the DNA sequence was identical between wildtype and no.7.

After the acclimatization, the 21 of segregants showed different growth curves in YNBG, indicating that the acclimatized BY4741 consisted of multiple strains (Figure 2). As the results showed, the wildtype had intraspecies diversity regarding glycerol assimilation that even derived from the purchased strain. As reported by Barnett et al. [18] and Swinnen et al. [17], *S. cerevisiae* strains over-cultivated in synthesized glycerol medium could have a high intraspecies diversity. The no.7 was picked up as the most glycerol-acclimatized strain in the group and was certified as the *S. cerevisiae*-derived strain. The no. 7 showed 0.0075 h^−1^ of *µmax* (*td*: 92.4 h) as the best score in the group; however, the value was quite low compared to *µmax* ≥ 0.08 h^−1^ of *S. cerevisiae* CBS 6412-13A [17]. Additionally, over 169 h of lag phase was long versus the lag phase ≤35 h of *S. cerevisiae* CBS 6412-13A [17]. Those results could suggest the possibility of insufficient acclimatization to the previously reported strain, meaning that the isolated strain no.7 was analyzed more in YNBG.

### 3.3. Time-Course Profiles of Biomass Production, Carbon Source Consumption, and Ethanol Production of Wildtype and No.7 in Each Medium under Aerobic Condition

**Figure 3:**
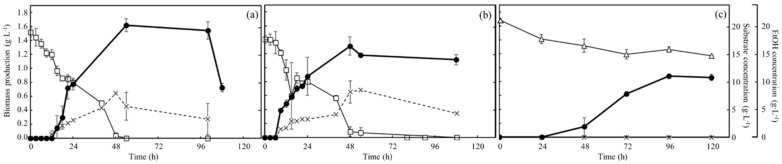
Time-course profiles of biomass production, carbon source consumption, and ethanol production of wildtype and no.7 in each medium under aerobic conditions. The figures show the profiles of (**a**) wildtype in SD, (**b**) no.7 in SD, and (**c**) no.7 in YNBG, under aerobic conditions. Symbols of the closed circle bold line, open square thin line, open triangle thin line, and cross-broken line correspond to biomass production (g·L^−1^), glucose concentration (g·L^−1^), glycerol concentration (g·L^−1^), and ethanol concentration (g·L^−1^), respectively. Glucose and glycerol concentrations are summarized as the substrate concentration in the figures. The growth profile depending on values of OD_600_ in YNBG was used as the secondary evaluation, and the values were converted into biomass production with the calibration curve.

The time-course profiles of concentrations of biomass production, substrates (glucose/glycerol), and ethanol under aerobic conditions are shown in Figure 3. Especially, the biomass profile of no.7 in YNBG was analyzed for a secondary evaluation. Regarding the wildtype in SD (Figure 3a), the biomass production was 1.6 g·L^−1^ at 54 h as the top value in those groups. The ethanol concentration reached 8.1 ± 0.4 g·L^−1^ at 48 h and gradually decreased to 3.4 g·L^−1^ at 100 h. Glucose was almost consumed in 48 h, and the timing was corresponding to the time points of the max values of biomass production and ethanol concentration. Depending on the analysis with OD_600_, the values of *µmax* and *td* of wildtype in SD were 0.15 h^−1^ and 4.5 h. Regarding no.7 in SD (Figure 3b), biomass production peaked at 1.3 ± 0.1 g·L^−1^ at 48 h. The ethanol concentration also peaked at 8.3~8.6 g·L^−1^ at 48~54 h, and then decreased to 4.6 g·L^−1^ by 108 h and glucose was almost completely consumed in 48 h. The *µmax* and *td*, calculated with OD_600_, were 0.15 h^−1^ and 4.6 h. Concerning no.7 in YNBG (Figure 3c), the biomass production was 0.9 ± 0.1 g·L^−1^ at 96 h as the maximum value in the condition. No ethanol concentration was detected until 120 h. Glycerol was consumed and the concentration was decreased from 21.4 to 15.1 g·L^−1^ in 72 h. No ethanol production was detected in YNBG and no correlation between glycerol consumption and ethanol production was confirmed. However, there was a correlation between biomass production and glycerol consumption, and the increase in biomass production reached the plateau at 96 h after stopping the consumption of glycerol at 72 h. The values of *µmax* and *td* of no.7 in YNBG were 0.034 h^−1^ and 20 h. After reaching the bottom value of glycerol once, the glycerol concentration was around 14.8~15.9 g·L^−1^ as the stationary value until 216 h.

The analyses of time-course profiles regarding concentrations of biomass, substrates (glucose and glycerol), and ethanol revealed the different properties of the strains and media (Figure 3). Culturing in SD, the *µmax* and the *td* of both strains of wildtype and no.7 in SD were 0.15 h^−1^ and 4.5~4.6 h. According to the findings and previous reports [19], the result displayed no difference in mitotic rate between no.7 and wildtype when glucose existed as a carbon source in the broth. However, there was a difference in biomass production between both strains, and the biomass production by no.7 could only reach around 80% of the one with the wildtype. The findings could indicate the change in metabolism in no.7 due to the acclimatization. Additionally, the results showing that the percent yield of ethanol by no.7 was approximately 95% at 54 h and higher than around 80% by wildtype might also mean a change in metabolism by the acclimatization. Both strains consumed the produced ethanol after glucose depletion at 48 h, indicating gluconeogenesis to secure an energy source. On the other hand, the *µmax* and the *td* of no.7 in YNBG were 0.034 h^−1^ and 20 h, indicating that those values were strongly enhanced 4.5-fold towards those (*µmax*: 0.0075 h^−1^; *td*: 92.4 h) of the isolate no.7 shown in Figure 2. Although the *µmax* of no.7 did not yet reach 0.08 h^−1^ of *S. cerevisiae* CBS 6412-13A [17], no.7 showed enough glycerol-assimilating activity comparing to wildtype. The no.7 gradually ingested glycerol in 72 h and biomass production increased until 96 h, meaning that no.7 could increase the biomass production with the glycerol taken-up in the cells after ingesting glycerol as a carbon source. After 96 h, no.7 did not consume glycerol and kept the biomass production for the time being. Additionally, the no.7 fermented no ethanol. Thus, no.7 could not aggressively perform the glycerol metabolism. Regarding the ethanol fermentation, no.7 might not control the redox balance in the cells. Generally, *S. cerevisiae* produces ethanol to recover NAD^+^. NAD^+^ is redacted to NADH when glyceraldehyde 3-phosphate reacts to d-1,3-bisphosphoglycerate in glycolysis [20,21]. The sum quantity of NAD^+^ and NADH is normally constant in a cell: without recovering NAD^+^, the metabolic reaction will stop after passing through glycolysis. In glycerol metabolism, ethanol could not be produced to regenerate NAD^+^ from NADH because of the difference in reduction power between glucose and glycerol, meaning that no.7 could not use glycerol as a carbon resource. Previously, Yu et al. constructed the strain overexpressing glycerol dehydrogenase and dihydroxyacetone kinase strongly to deliver glycerol to dihydroxyacetone phosphate as a component of glycolysis, resulting in the engineered strain being aerobically fermented ethanol with glycerol [22]. Therefore, although no ethanol fermentation by no.7 possibly implied little glycerol delivering to glycolysis, the possibility was low because of the biomass increase of no.7 with glycerol.

### 3.4. Time-Course Profiles of Carbon Source Consumption and Ethanol Production of Wildtype and No.7 in Each Medium under Anaerobic Condition

In the anaerobic broths of wildtype in SD, no.7 in SD, and no.7 in YNBG, the concentrations of glucose and glycerol as the substrate and ethanol as a kind of fermentation product were clearly detected by HPLC analyses (Table 1). After quantification, both strains of wildtype and no.7 in SD consumed 18.2 ± 0.5 and 21.6 ± 0.5 g·L^−1^ of glucose in 24 h, and those strains produced 8.5 ± 0.2 and 10.2 ± 0.5 g·L^−1^ of ethanol. The theoretical ethanol yield from 20 g·L^−1^ of glucose was 10.2 g·L^−1^, and wildtype and no.7 displayed 92.0 ± 4.7% and 92.4 ± 4.7% of the percent yield of ethanol in the culturing condition under the anaerobic condition in this study. On the other hand, no.7 in YNBG used no glycerol and fermented no ethanol.

Concerning wildtype in SD, no.7 in SD, and no.7 in YNBG, the anaerobic tendencies of using substrates (glucose and glycerol) and producing ethanol were evaluated (Table 1). In this study, wildtype and no.7 displayed around 8.5~10.2 g·L^−1^ and 92% as the yield and percent yield of ethanol. According to previous reports [23], the ethanol fermentation of BY4741 is 104.4 ± 3.1 g·L^−1^, and the percent yield of ethanol was 84.4–89.6%. Comparing our results with the previously reported ones, the values of ethanol fermentation in this study were better or higher. Therefore, with continuously adding glucose or increasing glucose concentration in the medium, wildtype and no.7 could possibly show the enhanced ethanol yield. On the other hand, no.7 performed no glycerol consumption, and no ethanol fermentation even under anaerobic conditions. The reason why no.7 did not ferment ethanol with glycerol might be that the strain could not control the redox balance in cells requiring surplus oxidation power towards NADH or NADPH during glycerol assimilation through these pathways. According to a previous report [24], nitrite was used to control redox balance in yeast cell as an oxidant and a reductant (balancer) and supported ethanol fermentation with a carbon source not derived from glucose. Therefore, no.7 could not control the redox balance and ferment no ethanol.

### 3.5. Distribution of Transcription Levels of Candidate Genes as Housekeeping Genes in Wildtype and No.7

**Figure 4:**
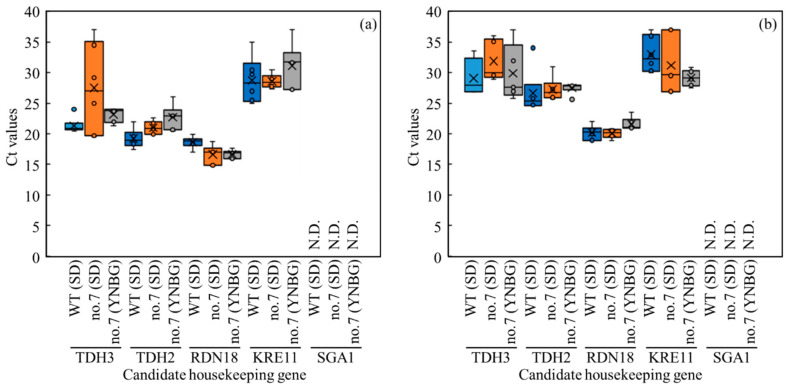
Distribution overview of transcription levels shown as Ct values of candidate genes as housekeeping genes in no.7 and wildtype. Boxplot representation of raw Ct values obtained from amplification curves (*n* = 6). Lower and upper boundaries of the box indicate the 25th and the 75th percentiles, the thin line within the box marks the median, and the whiskers (error bars) below and above the box indicate the 10th and 90th percentiles, respectively. CT values were obtained under (**a**) aerobic and (**b**) anaerobic conditions.

In this study, to precisely estimate the metabolic shifts of wildtype and no.7 under each culture condition in view of transcriptomics, the transcription levels of *TDH3*, *TDH2* (*glyceraldehyde-3-phosphate dehydrogenase*), *RDN18* (*structural constituent of ribosome*/*translation*), *KRE11* (*unknown*/*ER to Golgi vesicle-mediated transport*), and *SGA1* (*glucan 1,4-alpha-glucosidase*) as the candidate housekeeping genes [25,26,27] were evaluated by qPCR (Figure 4). In the results, excluding *SGA1*, delta Rn values of *TDH3*, *TDH2*, *RDN18*, *KRE11*, and *SGA1* were logistically increased even with the differences in wildtype and no.7 under different conditions, meaning those of transcription. Under aerobic conditions, the qPCR results showed the stable transcriptions of *TDH3*, *TDH2*, *RDN18*, and *KRE11* (Figure 4a). The cycle threshold (Ct) values of *RDN18* were 18.6 ± 1.0, 16.6 ± 1.6, and 16.6 ± 0.7 of the wildtype in SD, no.7 in SD, and no.7 in YNBG, and the values were stable in the candidate housekeeping gene group. The *SGA1* did not work well as a housekeeping gene in this study because of the unstable Ct values. In fact, in six time trials, the Rn value of *SGA1* was not detected four times in wildtype in SD, five times in no.7 in SD, and four times in no.7 in YNBG. The item of the Ct value of *SGA1* was displayed as not detected (N.D.) since the Rn values were not sufficient. Under anaerobic conditions, the qPCR results showed the stable transcriptions of *TDH3*, *TDH2*, *RDN18*, and *KRE11* (Figure 4b). The Ct values of *RDN18* were 20.2 ± 1.2, 20.2 ± 1.2, and 21.0 ± 0.1 of wildtype in SD, no.7 in SD, and no.7 in YNBG, and the values were also stable corresponding to the data under aerobic conditions. The *SGA1* also did not work as a housekeeping gene in this study with the unstable Rn values. Actually, in six time trials, the transcription of *SGA1* was not detected five times in wildtype in SD, five times in no.7 in SD, and in all trials of no.7 in YNBG. As with the aerobic condition, the item of the Ct value of *SGA1* was displayed as N.D.

Accurately performing qPCR requires the selection of housekeeping genes suited to each experiment to avoid huge scattering of the transcript level; therefore, various housekeeping genes were selected depending on each experiment so far (Figure 4). The genes of *TDH3*, *TDH2*, *RDN18,* and *KRE11* were steadily transcribed. Especially, the transcription of *RDN18* was stable in all strains, media, and aerobic/anaerobic conditions; therefore, this *RDN18* was used as the housekeeping gene. *TDH3* and *TDH2* were isogenes at the boundary between the preparatory phase and pay-off phase in glycolysis. TDH3 and TDH2 were important enzymes for generating reduction power in glycolysis, estimating that the isogenes could be regularly transcribed in all conditions. The transcription regarding *RDN18* was estimated as below: maintaining the translation of genes is required to save lives so the stable construction of ribosomes at a place of translation is needed; *RDN18* was a structural constituent of ribosome/translation, meaning that *RDN18* could be stably transcribed without huge scattering in any conditions. Additionally, the Ct value of *RDN18* was the lowest in the candidates under aerobic and anaerobic conditions, meaning of the highest transcription level. The result could indicate that *RDN18* was an essential component for maintaining biological activity and showing the requirement of stable and high-level transcription. These findings are in accordance with the low Ct value of *RDN18* without huge scattering derived from the previous report by Teste et al. [25]. *KRE11* was unknown/ER to Golgi vesicle-mediated transport. Therefore, the level of transcription could depend on the cell needs of proteins requiring transportation, meaning the scattering of the levels. The reason why the transcription of *SGA1* did not appear could be that there was no need to hydrolyze terminal 1,4-linked alpha-D-glucose residues from non-reducing ends of polysaccharide chains with the release of beta-glucose under those conditions. Depending on those results regarding the Ct values, it was finally decided to use *RDN18* as the housekeeping gene.

### 3.6. Relative Quantification of mRNA of Wildtype and No.7 in SD and in SD/YNBG under Aerobic Condition

**Figure 5:**
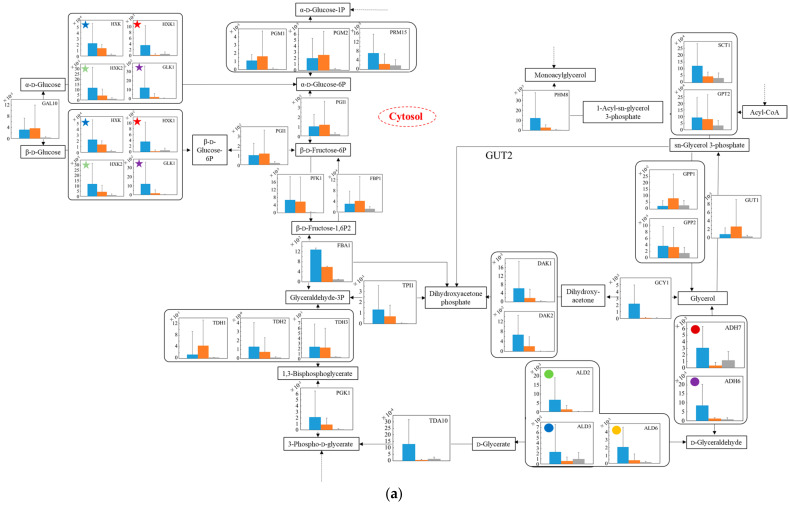

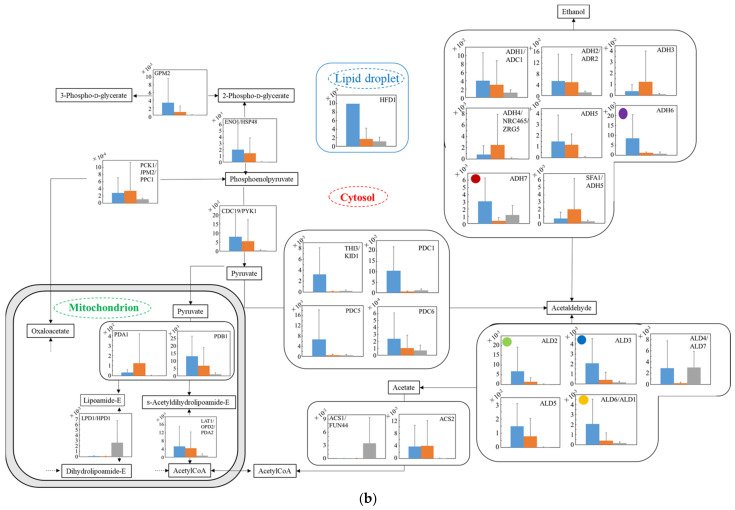

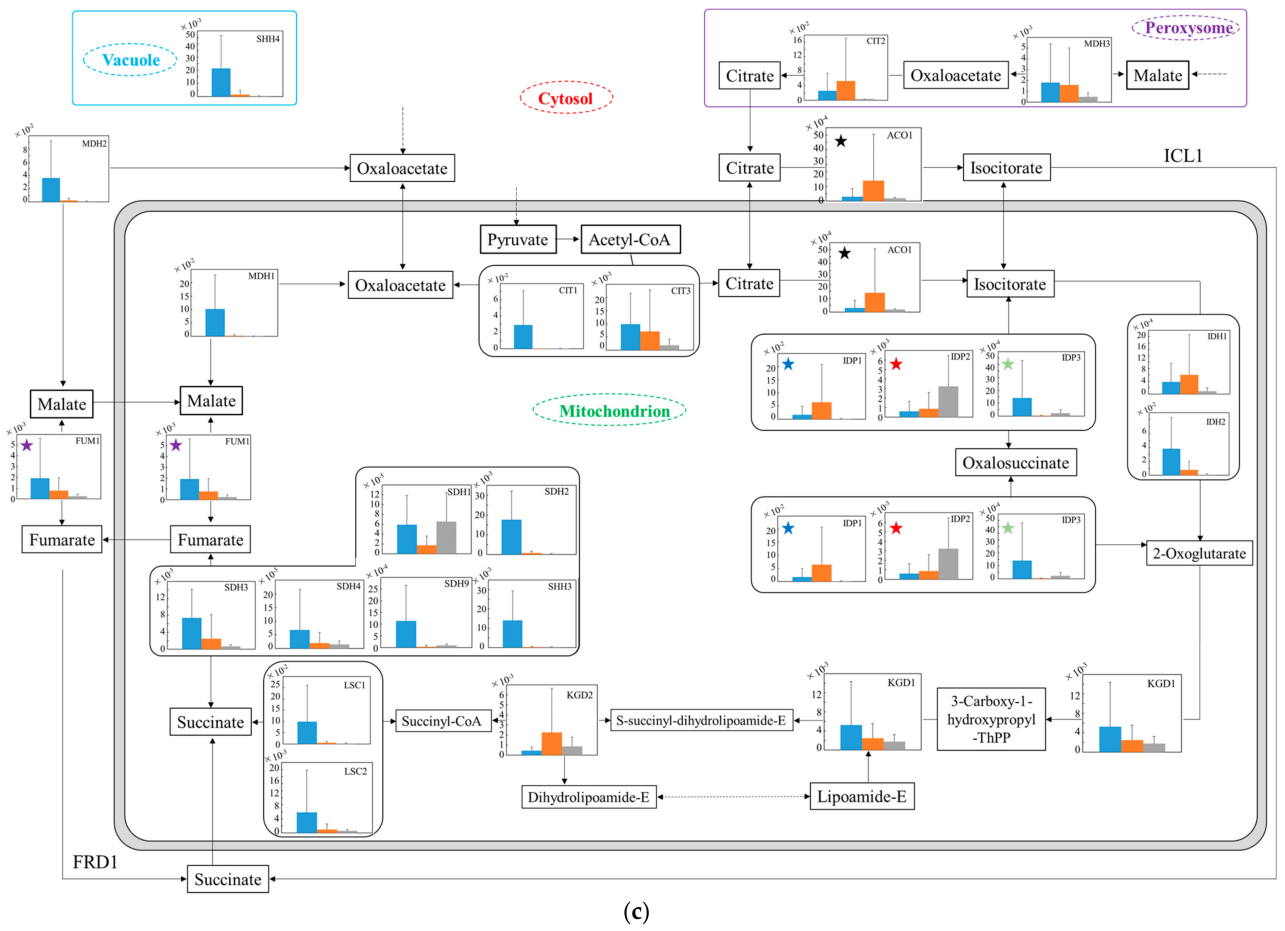
Relative quantification of mRNA of wildtype and no.7 in SD and in SD/YNBG under aerobic conditions. Shown data are the relative mRNA levels normalized by the level of *RDN18* as a housekeeping gene and by level of each gene of wildtype and no.7 under different media (SD and YNBG) at the exponential phase. The relative mRNA levels of wildtype in SD, no.7 in SD, and no.7 in YNBG are shown as blue, orange, and gray bars, respectively. Error bars indicate the SD of six replicate experiments (*n* = 6). The metabolic map is divided into three parts mainly including the (**a**) glucose–glycerol pathway, (**b**) pyruvate-ethanol pathway, and (**c**) TCA cycle. The same-colored star marks in same figures mean transcription levels of the same enzymes; same-colored circles marks in (**a**,**b**) mean transcription levels of the same enzymes, especially ALDs and ADHs.

To estimate the difference in carbon metabolism in each strain under aerobic conditions (Figure 5), the transcription levels were analyzed in the glucose–glycerol pathway (Figure 5a), pyruvate–ethanol pathway (Figure 5b), and tricarboxylic acid (TCA) cycle (Figure 5c). In Figure 5a, regarding the glucose metabolism in glycolysis, the transcription levels from glucose to 3-phospho-d-glycerate of no.7 in YNBG were mostly low compared to those of both strains in SD. Regarding the metabolism from glycerol to glycolysis, in spite of the strain difference, the transcription levels of *GCY1* and *DAK1*/*DAK2* via dihydroxyacetone in SD were higher than the ones in YNBG. On the other hand, the transcription levels of *ADH7* and *ALD3* via d-glyceraldehyde even in YNBG were similar to those in SD. Additionally, via sn-glycerol 3-phosphate, the transcription level of *GUT1* of no.7 in YNBG was not improved. In Figure 5b, as for the metabolic production from pyruvate to ethanol, the transcription levels of *THI3*, *PDC6*, *PDC5*, and *PDC1* especially of wildtype in SD were higher than the ones of no.7 in both media. In no.7, although the transcription levels of *LPD1* regarding the metabolism from pyruvate to oxaloacetate in YNBG were higher than the ones in SD, the transcription levels of *LAT1* regarding metabolism from pyruvate to acetyl CoA in SD were higher than the ones in YNBG. Concerning the metabolism from acetyl CoA to acetate, the switching between *ACS2* and *ACS1* was detected depending on the carbon sources of glucose and glycerol. In Figure 5c, from isolate to 2-oxoglutarate in the TCA cycle, the transcription level of *IDP2* of no.7 in YNBG was higher than the others. On the other hand, *IDH1*/*IDH2* in SD were highly translated compared to in YNBG. Similarly, between fumarate and succinate in the TCA cycle, although the transcription levels of wildtype were higher than the others, the transcription level of *SDH1* of no.7 was improved similar to the case of wildtype. Regarding the later metabolism of the TCA cycle, in SD, the transcription levels related to enzymes from succinate to citrate in wildtype trended to be higher than the ones in no.7. The transcription level of *MDH2* in wildtype was especially higher than the others in no.7.

The transcription levels were analyzed to estimate the metabolic differences between wildtype and the glycerol-acclimatized strain no.7 under aerobic conditions (Figure 5). In Figure 5a, in spite of the strain differences, the transcription levels from glucose to 3-phospho-D-glycerate in YNBG were mostly lower than the ones in SD. In YNBG under aerobic respiration, no.7 did not need to metabolize glucose so that the strong transcriptions in the preparatory phase of glycolysis as the upper stream from 1,3-bisphosphoglycerate would be not required for energy production. Additionally, regarding the metabolism from glycerol to glycolysis in YNBG, the transcription levels of *GCY1* and *DAK1*/*DAK2* were mostly depressed and also the level of *GUT1* was not improved. Previous reports by Ho et al. and Islam et al. proved that the activated GUT and DAK by genetic engineering improved glycerol use in *S. cerevisiae* [28,29]; however, those transcription levels were not upregulated in no.7. The reason why no.7 can utilize glycerol was not based on the activities of those pathways of glycerol intake. On the other hand, the transcription levels of *ADH7* and *ALD3* even in YNBG were similar to the ones in SD regardless of strain difference. At least, in no.7, glycerol could also flow into glycolysis via D-glycerate under aerobic conditions so that the strain could use glycerol. In SD, in the pathway of glycerol to glycolysis, higher transcription levels of wildtype than of no.7 could indicate the recovery of NAD^+^ (e.g., reactions by DAK1 and DAK2) to use glucose in glycolysis. The findings might also show a correlation with the different biomass production between wildtype and no.7. In Figure 5b, in SD, the results implied that the metabolic activity from pyruvate to acetaldehyde in wildtype was higher than in no.7. Indeed, in SD, the ethanol production by wildtype was higher than by no.7, and the values were 5.5 ± 0.2 and 4.2 ± 0.4 g·L^−1^ by wildtype and no.7 at 40 h. There were probably few possibilities that the enzymatic activity of ADH decreased the metabolic activity due to a lot of the ADH isozymes existing and the activity remaining from acetaldehyde to ethanol. Therefore, the metabolic activity from pyruvate to acetaldehyde in no.7 could be depressed in ethanol fermentation. The transcription levels downstream from pyruvate were changed depending on the carbon sources, due to metabolic flow not escaping into the TCA cycle. In the case of using glucose as a carbon source, the production of acetate could be enhanced by activated LAT1; using glycerol, the production of dihydroxy lipoamide could be promoted by acerated LPD1. In Figure 5c, the transcription levels related to enzymes from succinate to citrate in the TCA cycle in wildtype trended to be higher than in no.7. By nature, *S. cerevisiae* is a facultative anaerobic organism, and the TCA cycle is activated to prepare oxidative phosphorylation under aerobic conditions using glucose aggressively. From isolate to 2-oxoglutarate in the TCA cycle, no.7 could switch the transcription of *IDP2* or *IDH1*/*IDH2* depending on the media. The results might indicate the change in metabolic flow with carbon source, meaning that the carbon metabolic flow from isocitrate could pass though oxalosuccinate in YNBG and directly to 2-oxoglutarate in SD. Similarly, between fumarate and succinate in the TCA cycle, no.7 could preferably use SDH1 rather than the other enzymes in YNBG. The TCA cycle activities in no.7, which was acclimatized with glycerol, might decrease comparing to the ones in wildtype. In fact, as shown in Figure 3, no.7 displayed lower biomass production than wildtype, and there was a possibility of decreasing energy producing efficiency. The higher transcription level of *MDH2* in wildtype could indicate that the metabolic reaction from malate to oxaloacetate was supported by cytosolic MDH2 not only in the TCA cycle.

The total trait of transcription levels no.7 was compared to that of another glycerol-assimilating strain. Kawai et al. also obtained glycerol-assimilating strains from *S. cerevisiae* by repeated subcultivation in a glycerol-based medium [30]. The transcription levels of the strains in glycerol-based medium were compared to those in glucose-based media, resulting in the transcription levels of *TDH2*, *ENO2*, and *CDC19* as key genes being downregulated in glycolysis; the levels of most genes were upregulated excluding *FUM1* in the TCA cycle. Especially, in the TCA cycle, the transcription levels regulating the metabolic reaction between succinate and fumarate were enhanced. In the ethanol-producing pathway, the levels regulating the reaction between acetaldehyde and ethanol were maintained or increased, excluding *ADH1*. On the other hand, in this study, no.7 also depressed the transcription levels of *TDH*, *ENO*, and *CDC* so that glycerol-assimilating strains might decrease the activity in glycolysis. In the TCA cycle in no.7, although the transcription levels of enzymes regulating the reaction between succinate and fumarate showed few differences, one of *SDH1* displayed the increment trait. Additionally, between isocitrate and 2-oxiglutarate, the trait of shift in the pathway might also be indicated by the increased transcription level of IDP2. Unlike glycolysis, the TCA cycle may try to maintain a metabolic response. In the metabolic reaction between acetaldehyde and ethanol in no.7, although the trait of maintained or decreased transcription levels was confirmed, the transcription level of *ADH7* was enhanced. The results might indicate that no.7 tried to maintain the reaction activity similar to the glycerol-assimilating strains picked up by Kawai et al. Therefore, no.7 showed similarities to another glycerol-assimilating strain from *S. cerevisiae* in the transcription tendency of the genes related to glycerol assimilation; however, the transcription tendency of each gene was different, indicating the diversity of *S. cerevisiae*.

### 3.7. Relative Quantification of mRNA of Wildtype and No.7 in SD and in SD/YNBG under Anaerobic Condition

**Figure 6:**
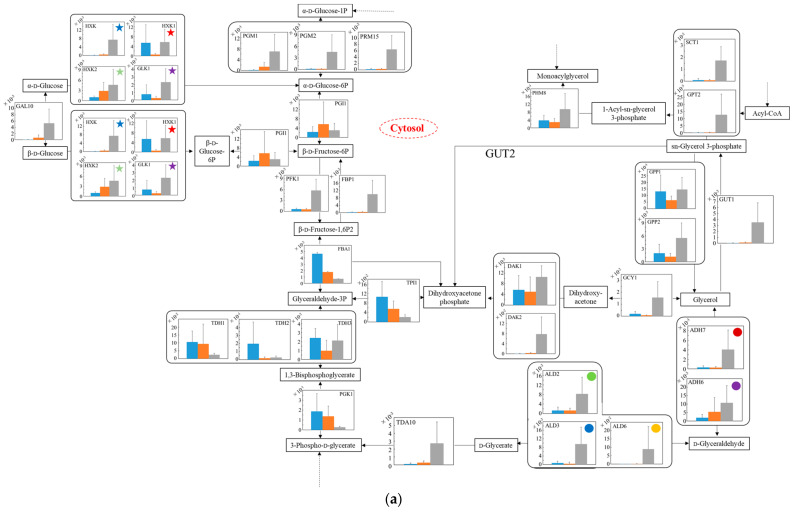

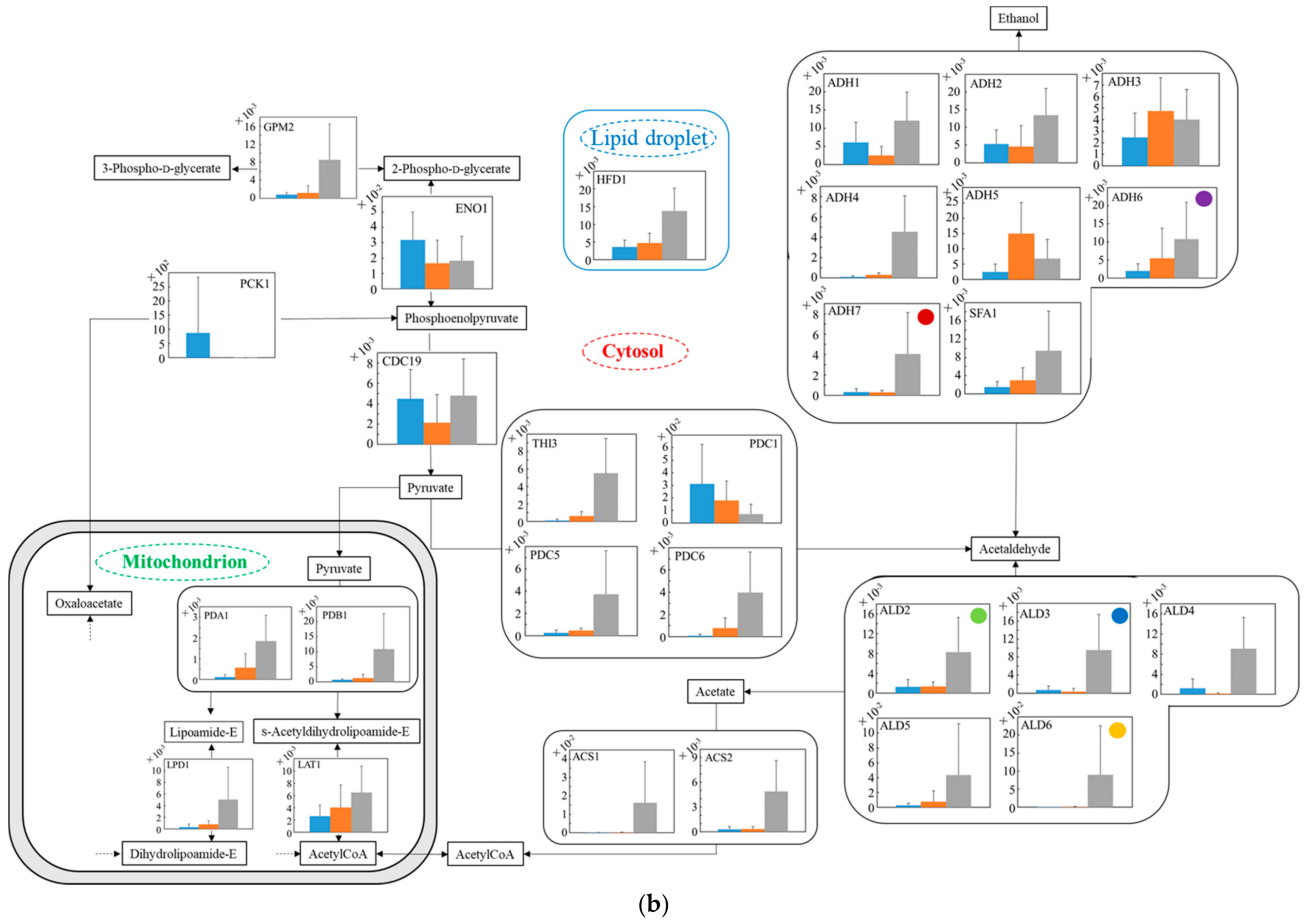

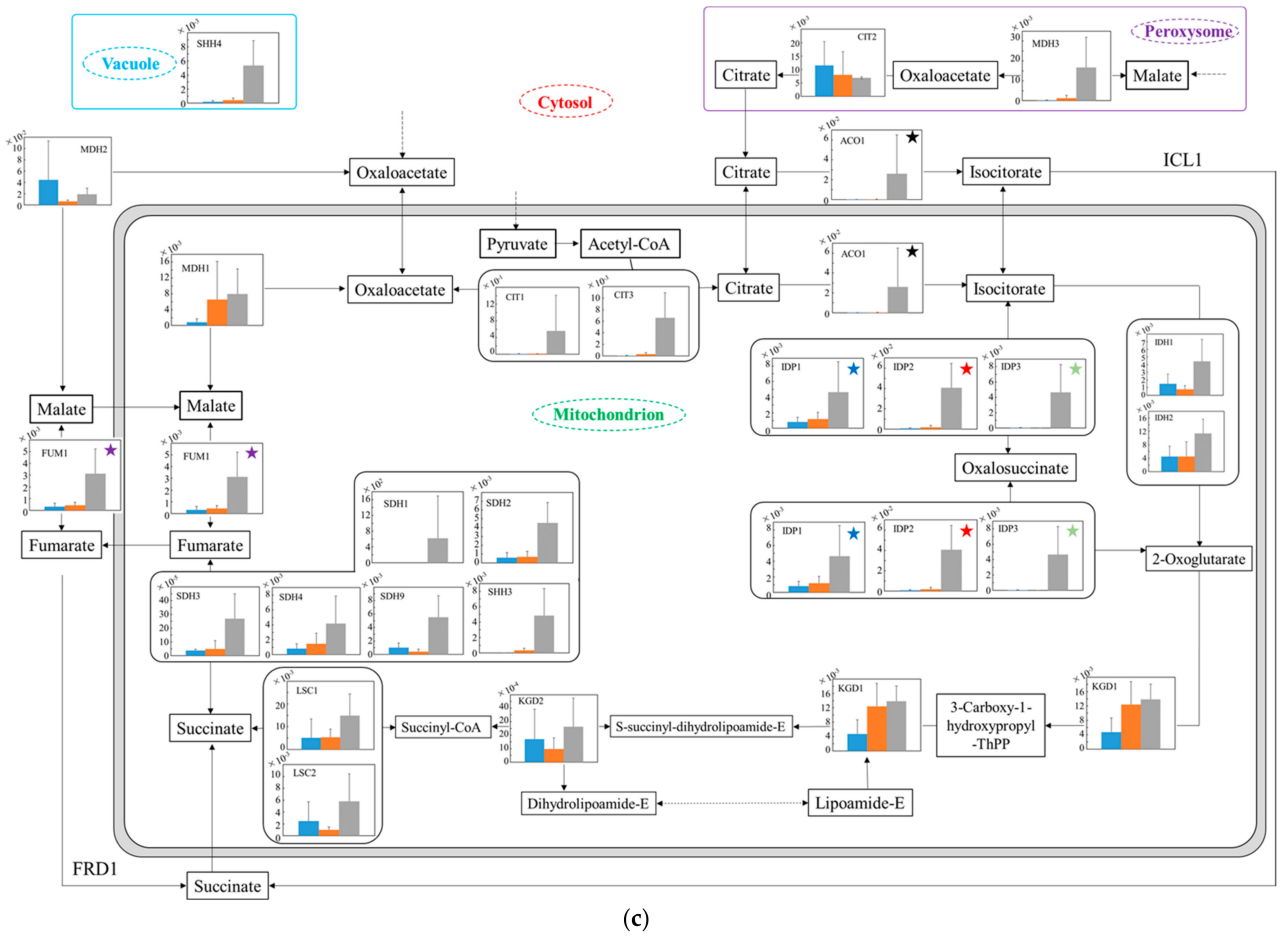
Relative quantification of mRNA of wildtype and no.7 in SD and in SD/YNBG under anaerobic conditions. Shown data are the relative mRNA levels normalized by level of *RDN18* as a housekeeping gene and by level of each gene of wildtype and no.7 under different media (SD and YNBG) at 24 h. The relative mRNA levels of wildtype in SD, no.7 in SD, and no.7 in YNBG are shown as blue, orange, and gray bars, respectively. Error bars indicate the SD of six replicate experiments (*n* = 6). The metabolic map is divided into three parts mainly including the (**a**) glucose–glycerol pathway, (**b**) pyruvate-ethanol pathway, and (**c**) TCA cycle. The same-colored star marks in the same figures mean transcription levels of the same enzymes; same-colored circle marks in (**a**,**b**) mean transcription levels of the same enzymes, especially ALDs and ADHs.

Estimating the carbon metabolism around glycolysis and the TCA cycle in each strain under anaerobic conditions (Figure 6), the transcription levels were also analyzed in the glucose-glycerol pathway (Figure 6a), pyruvate-ethanol pathway (Figure 6b), and TCA cycle (Figure 6c). In Figure 6a, in regard to the metabolism from glycerol to glycolysis, even in YNBG, the transcription levels through the pathway via dihydroxyacetone in no.7 were similar or superior to the ones in the wildtype. The phenomena under anaerobic conditions were different to those under aerobic conditions. Especially, the transcription levels of *DAK2* and *GCY1* of no.7 in YNBG were higher than the others. Relating to via dihydroxyacetone phosphate, the transcription level of *GUT1* of no.7 in YNBG was also superior to the others. On the other hand, in YNBG, all transcription levels through the pathway via D-glyceraldehyde in no.7 were higher than the others. In spite of the difference in wildtype and no.7, the level of *PGK* in SD was superior to that in YNBG. In Figure 6b, regarding the metabolism from pyruvate to ethanol, regardless of the strain differences, the transcription levels relating to enzymes in YNBG were almost the same and/or higher than the ones in SD. In Figure 6c, regarding the later metabolism in TCA cycle, in spite of the strain differences in wildtype and no.7, the transcription levels of enzymatic genes from succinate to citrate in YNBG tended to be higher than the ones in SD. The phenomena under anaerobic conditions were also different to those under aerobic conditions. Regarding the metabolic reaction between malate and oxalate, *MDH2* of wildtype in SD, *MDH1*/*MDH2* of no.7 in SD, and *MDH1*/*MDH2*/*MDH3* of no.7 in YNBG were detected as well-balanced transcription.

Additionally, under anaerobic conditions, the transcription levels were analyzed to estimate the metabolic differences between wildtype and the glycerol-acclimatized strain no.7 (Figure 6). In Figure 6a, the transcription levels from glycerol to glycolysis were activated in YNBG. Under anaerobic conditions, the enhanced expression of enzymes related to intake of glycerol could be reinforced to move glycerol into glycolysis. Especially, the significant enhancement of *GUT1*, which was not detected under aerobic conditions, could significantly support glycerol assimilation via dihydroxyacetone. However, provided that the transcription level of *PGK* in glycolysis was not enough in no.7 in YNBG, the main carbon metabolic flow might be the pathway via D-glyceraldehyde. In Figure 6b, in YNBG, the transcription levels related to ethanol fermentation under anaerobic conditions were mostly enhanced rather than under aerobic condition, and the level of *ADH4* was especially improved. Although the higher expression would promote the expression of the ethanol-producing enzymes in no.7 in YNBG, the ethanol production was not detected. Therefore, with glycerol as a carbon source, the redox balance in cells would be difficult to control: especially the oxidation of NADH through acetaldehyde to ethanol would be difficult, resulting in no ethanol production. In Figure 6c, from succinate to citrate in the TCA cycle, the transcription levels of no.7 tended to be higher than those of wildtype, and the phenomena were opposite under aerobic conditions. The no.7 strain could activate the transcription to create energy under anaerobic conditions because the strain could utilize glycerol. In the metabolic reaction relating to malate and oxalate, the use of enzymes could be different depending on the condition. As a result, wildtype in SD, no.7 in SD, and no.7 in YNBG could use mainly MDH2, MDH1/MDH2, and MDH1/MDH2/MDH3, respectively. In *S. cerevisiae*, cytosolic MDH2 could originally play a main role in the reaction, and MDH1 and MDH3 were possibly able to join to the reaction by glycerol acclimatization. In the reaction, NADH can be reduced by metabolic flow from oxalate to malate so that the transcription levels relating to the genes might be improved to obtain reduction power.

## 4. Conclusions

In this study, 21 glycerol-assimilating strains were successfully picked up from *S. cerevisiae* BY4741. The 21 strains showed different growth curves in YNBG indicating intraspecies diversity even though they were segregants. The no.7, which showed the highest relative growth rate in the 21 strains, was picked up to analyze the glycerol assimilation system of *S. cerevisiae*. Although no.7 displayed no difference in *µmax* and the *td* for wildtype when glucose existed as a carbon source in the broth, there was a difference in biomass production and the percent yield of ethanol. The results could indicate the change in metabolism in no.7 by acclimatization. In the glycerol medium, no.7 could increase the biomass production with glycerol taken up in the cells after ingesting glycerol as a carbon source. After the process, no.7 did not consume glycerol for the time being nor ferment ethanol. The reason why no.7 did not ferment ethanol with glycerol might be that the strain could not control the redox balance in cells requiring surplus oxidation power towards NADH or NADPH during glycerol assimilation through these pathways. To perform qPCR, transcription levels of *TDH3*, *TDH2*, *RDN18*, *KRE11*, and *SGA1* were evaluated for use as housekeeping genes. The transcription of *RDN18* was stable in all strains, media, and aerobic/anaerobic conditions because the *RDN18* was deeply related to construction of ribosomes. Therefore, this *RDN18* was used as the housekeeping gene. Under aerobic conditions, without the strain differences, the transcription levels from glucose to 3-phospho-D-glycerate in YNBG were mostly lower than the ones in SD. Additionally, regarding the metabolism from glycerol to glycolysis in YNBG, the transcription levels of *GCY1* and *DAK1*/*DAK2* were mostly depressed; *GUT1* was not improved; *ADH7* and *ALD3* even in YNBG were similar to the ones in SD regardless of strain difference. Especially, *GCY1* was depressed as a different property of the no.7 strain to wildtype. At least, in no.7, glycerol could also flow into glycolysis via D-glycerate under aerobic conditions so that the strain could use glycerol. From isolate to 2-oxoglutarate in the TCA cycle, no.7 could switch the transcription of *IDP2* or *IDH1*/*IDH2* depending on the media. Those results might indicate the change in metabolic flow with carbon source, meaning that the carbon metabolic flow from isocitrate could pass though oxalosuccinate in YNBG and directly to 2-oxoglutarate in SD. The TCA cycle might attempt to maintain a metabolic response not like glycolysis. Therefore, no.7 showed similarities to other glycerol-assimilating strains; however, the different transcription tendency of each gene could indicate the diversity of *S. cerevisiae*. Additionally, under anaerobic conditions, the transcription levels of no.7 from glycerol to glycolysis were activated in YNBG. Especially, the significant enhancement of the *GUT1* was not detected under aerobic conditions. The result could significantly support glycerol assimilation via dihydroxyacetone. In YNBG, the transcription levels related to ethanol fermentation were mostly enhanced and *ADH4* was especially improved even without ethanol production. Therefore, with glycerol as a carbon source, the redox balance in cells would be difficult to control: especially the oxidation of NADH through acetaldehyde to ethanol would be difficult, resulting in no ethanol production. In the TCA cycle, our results showed the possibility to change the enzymes for the metabolic reaction relating to malate and oxalate depending on the condition. In *S. cerevisiae*, cytosolic MDH2 could originally play a major role in the reaction, and MDH1 and MDH3 could possibly join the glycerol acclimatization. In the reaction, NADH can be reduced by metabolic flow from oxalate to malate, resulting in the transcription levels possibly being improved to obtain reduction power.

This study estimated the metabolic switching by qPCR with glycerol acclimatization of *S. cerevisiae*, and endowed knowledge to use glycerol as a carbon source by *S. cerevisiae*. For instance, this research displayed that low *PGK1* transcription could be related to the metabolic rate-limiting step in a glycerol-based medium. Improvement in *PGK1* transcription is possible to enhance the use of waste glycerol with *S. cerevisiae*, which has robustness and can be genetically modified. The resource recovery of wasted glycerol will support the decreasing environmental stresses.

## Figures and Tables

**Table 1:** Time-course profiles of carbon source consumption and ethanol production of wildtype and no.7 in each medium under anaerobic condition. Experimental replication was undertaken three times, respectively.

	Wildtype in SD		No.7 in SD		No.7 in YNBG	
	0 h	24 h	0 h	24 h	0 h	24 h
Glucose (g·L^−1^)	20.8 ± 1.1	2.6 ± 0.6	22.3 ± 1.2	0.7 ± 0.7		
Glycerol (g·L^−1^)					21.7 ± 2.5	22.1 ± 0.2
Ethanol (g·L^−1^)	0.0 ± 0.0	8.5 ± 0.2	0.0 ± 0.0	10.2 ± 0.5	0.0 ± 0.0	0.0 ± 0.0

## Supplementary Materials

The following supporting information can be downloaded at: https://www.mdpi.com/article/10.3390/microorganisms10061173/s1, Supplementary Table S1 is gene information used in analyses of qPCR. All data were mainly provided by Alliance of Genome Resources, November 2021 and support data were obtained from additional references [31,32,33,34,35,36,37,38,39,40]. Supplementary Table S2 is primer-pair information of each enzyme gene prepared for qPCR.
