# Supplementary material for: Estimation of Carbon Metabolism in Saccharomyces cerevisiae Acclimatized to Glycerol Assimilation with Quantitative PCR
Source: Microorganisms. 2022 Jun 7;10(6):1173. doi: 10.3390/microorganisms10061173 (PMC9231053; doi:10.3390/microorganisms10061173)
Supplement: Supplementary file 1 [file microorganisms-10-01173-s001.zip › microorganisms-1733074-supplementary.pdf]

Supplementary Materials

Supplemental Table S1. Gene information. All data were mainly provided by Alliance of Genome Resources, Nov 2021 and support data were obtained from additional references.

Gene	Location	Other Expression	Additional Reference
PGM1	cytosol		
PGM2	cytosol	*GAL5*	
PRM15	cytosol; nucleus	*PGM3*	
PGI1	mitochondrion	*CDC30*	(1)
HXK	cytosol; mitochondrion		
HXK1	mitochondrion		(2)
HXK2	cytosol; mitochondrial membrane; nucleus	*HEX1*, *HKB*, *SCI2*	
GLK1	cytosol; plasma membrane	*HOR3*	
GAL10	cytosol		
PFK1	mitochondrion		(3)
FBP1	cytosol; periplasmic space	*ACN8*	
FBA1	cytosol; mitochondrion	*LOT1*	
TPI1	mitochondrion		(4)
TDH1	cell wall; lipid droplet; mitochondrion	*GLD3*	(2)
TDH2	cell wall; lipid droplet; mitochondrion	*GLD2*	(2)
TDH3	cell wall; lipid droplet; mitochondrion	*GLD1*, *HSP35*, *HSP36*, *SSS2*.	(2)
PGK1	mitochondrion		
TDA10	cytosol; nucleus		
ALD2	cytosol		(5),(6)
ALD3	cytosol		(7)
ALD4	mitochondrion; cytosol		(5),(8)
ALD5	mitochondrion; cytosol		(5),(6)
ALD6	mitochondrion; cytosol		(5)
HFD1	lipid droplet		
GCY1	cytosol; nucleus		
DAK1	cytosol		
DAK2	cytosol		
GUT1	mitochondrion; cytosol		(9)
GPP1	cytosol		
GPP2	cytosol; nucleus	*HOR2*	
GPT2	endoplasmic reticulum; lipid droplet	*GAT1*	
SCT1	endoplasmic reticulum; lipid droplet	*GAT2*	
PHM8	cytosol; nucleus		
GPM2	cytosol		

ENO1	fungal-type vacuole; mitochondrion; cytosol	*HSP48*	(10)
CDC19	cytosol	*PYK1*	
PCK1	cytosol	*JPM2, PPC1*	
PDA1	mitochondrial nucleoid		
PDB1	mitochondrial nucleoid		
THI3	nucleus	*KID1*	
PDC1	cytosol; nucleus; replication compartment		
PDC5	nucleus		
PDC6	cytoplasm	*RHR2*	
LPD1	mitochondrion	*HPD1*	
LAT1	mitochondrion	*ODP2, PDA2*	
ACS1	cytosol	*FUN44*	
ACS2	cytosol; nucleolus		
HFD1	lipid droplet		
ADH1	cytoplasm; replication compartment; cytosol	*ADC1*	
ADH2	cytosol	*ADR2*	
ADH3	mitochondrial matrix; cytosol		
ADH4	mitochondrion; cytosol	*NRC465, ZRG5*	
ADH5	cytoplasm; nucleus		
ADH6	cytosol		
ADH7	cytosol		
SFA1	mitochondrion	*ADH5*	
CIT1	mitochondrion	*LYS6*	
CIT2	peroxisome		
CIT3	mitochondrion		
ACO1	cytosol; mitochondrion	*GLU1*	
IDP1	mitochondrion		
IDP2	cytosol		
IDP3	peroxisome		
IDH1	mitochondrion		
IDH2	mitochondrion		
KGD1	mitochondrion	*OGD1*	
LPD1	mitochondrion	*HPD1*	
KGD2	mitochondrion		
LSC1	mitochondrion		
LSC2	mitochondrion		

SDH1	mitochondrion	
SDH2	mitochondrion	*ACN17*
SDH3	mitochondrion	*CYB3*, *YKL4*
SDH4	mitochondrion	*ACN18*
SDH9	mitochondrion	
SHH3	mitochondrion	
SHH4	fungal-type vacuole	
FUM1	cytosol; mitochondrion; nucleus.	
MDH1	mitochondrion	
MDH2	cytosol; nuclear periphery	
MDH3	peroxisome	
PCK1	cytosol	*JPM2*, *PPC1*

Supplemental References

(1) Papapetridis I, Verhoeven MD, Wiersma SJ, Goudriaan M, van Maris AJA, Pronk JT. Laboratory evolution for forced glucose-xylose co-consumption enables identification of mutations that improve mixed-sugar fermentation by xylose-fermenting *Saccharomyces cerevisiae*. 2018. FEMS Yeast Res 18:foy056.

(2) Thakur SS, Geiger T, Chatterjee B, Bandilla P, Fröhlich F, Cox J, Mann M. Deep and highly sensitive proteome coverage by LC-MS/MS without prefractionation. 2011. Mol Cell Proteomics 10:M110.003699.

(3) Petelenz-Kurdziel E, Kuehn C, Nordlander B, Klein D, Hong K-K, Jacobson T, Dahl P, Schaber J, Nielsen J, Hohmann S, Klipp E. Quantitative analysis of glycerol accumulation, glycolysis and growth under hyper osmotic stress. 2013, PLoS Comput. Biol. 9:e1003084.

(4) Marta V, Kostyantyn V. Metabolic engineering for high glycerol productionby the anaerobic cultures of *Saccharomyces cerevisiae*. 2016. apply microbial biotechnol 101:4403-4416.

(5) Daran-Lapujade P, Jansen MLA, Daran JM, van Gulik W, de Winde JH, Pronk JT. Role of transcriptional regulation in controlling fluxes in central carbon metabolism of *Saccharomyces cerevisiae*. A chemostat culture study. 2004. J Biol Chem 279:9125-9138.

(6) Yu M, Juwono N.K.P, Foo J.L, Leong S.S, Chang M.W, Metabolic engineering of *Saccharomyces cerevisiae* for the overproduction of short branched-chain fatty acids. 2016. Meta. Eng 34:36-43.

(7) Bakker BM, Bro C, Kotter P, Luttik MAH, Dijken JP, Pronk JT. The Mitochondrial alcohol Dehydrogenase Adh3p Is Involved in a Redox Shuttle in Saccharomyces cerevisiae. 2000. ASM 182:4730-4737.

(8) Hoang P.T.N, Ko ja K, Gong G, Um Y, Lee S.M. Genomic and phenotypic characterization of a refactored xylose-utilizing *Saccharomyces cerevisiae* strain for lignocellulosic biofuel production. 2018. Biotechnol Biofuel Bioproducts 11:268.

(9) Costenoble R, Valadi H, Gustafsson L, Niklasson C, Franzén CJ. Microaerobic glycerol formation in *Saccharomyces cerevisiae*. Yeast 16: 1483-1495.

(10) Norbeck J, Blomberg A. Metabolic and regulatory changes associated with growth of *Saccharomyces cerevisiae* in 1.4 M NaCl. Evidence for osmotic induction of glycerol dissimilation via the dihydroxyacetone pathway.1997. J Biol Chem 272:5544-5554.

Supplemental Table S2. Primer-pairs of each enzyme gene were prepared for qPCR.

Gene		Primer Sequences (5'→3')
TDH2	F	cggtagatacgctggtgaagtttc
	R	tggaagatggagcagtgataacaac
TDH3	F	tttcccacgatgacaagcac
	R	gaagcccatggcaagttagc
RDN18	F	aactcaccaggtccagacacaataagg
	R	aaggtctcgttcgttatcgcaattaagc
KRE11	F	aactggttctgttacccaaatcaactcaac
	R	aacgcttcaatgtgacttctgtttccc
SGA1	F	tccaaacggatatttcctgggtggtactgag
	R	gcatgatctattgtgtttacattagcgggtag
PGM1	F	tgatcctgccaaagcatacg
	R	ttgcgctgtttcgctaagaa
PGM2	F	gcctggtacctctggtttgc
	R	ccatcaccaccgacaacaag
PRM15	F	aagaggtcaccgctctctgc
	R	accagcagtgccaaactgaa
PGI1	F	acttcacccaaaccccattg
	R	tcgaatggagcaaccaaatg
HXK	F	aggggaggtagttgcaatgg
	R	tccggtgaaaggaagattgg
HXK1	F	attccaatgattcccggttg
	R	ccgctcaacttgaccaacac
HXK2	F	aaattttgcgtttggccttg
	R	tgtccatgacgaaaggcttg
GLK1	F	gccggtcatcggatgtattt
	R	gacccccattcgacattgat
GAL10	F	tgaaatggcagaccgagttg
	R	gcctcgacaccccttaactg
PFK1	F	actcccgtgcctttgttgtt
	R	atcggcaccggtagcaatac
FBP1	F	cgcctcaaaaggccatctac
	R	tgtctgcttggggttgtttg
FBA1	F	ggctttgcacccaatctctc
	R	aaagcgatgtcaccagcgta
TPI1	F	ggtaccggtttggctgctac
	R	gctaccgttagcggaaccac

TDH1	F	atggtccatcccacaaggac
	R	ggcaagaccttaccgacagc
PGK1	F	attggtggtggtgacactgc
	R	agcaacacctggcaattcct
TDA10	F	gcgcaacccagaaatcaaat
	R	gcacttgttcgtcggtcatc
ALD2	F	tcattcccccaaccatcttc
	R	gcgagcccgtagcaagtatc
ALD3	F	tcattcccccaaccatcttc
	R	gcgagcccgtagcaagtatc
ALD4	F	aaccaacggggttgttcatc
	R	gcctcttccacatcgtcctc
ALD5	F	aaggtcgctgccgatactgt
	R	cgcagcaaacttcaccagag
ALD6	F	tgaatgggctacccaagacc
	R	gtaacatccccacgggctaa
HFD1	F	gcgcacaaaaggaaaagctc
	R	ggcgtaacccccacttgtta
GCY1	F	tggcagtcgaaagagaacga
	R	ttgatggcttgaccgacttg
DAK1	F	aggcggtaaaagctgcagag
	R	tgaatcgccgacatacgaag
DAK2	F	ctgatcgatgctctgcaacc
	R	ttcggcaccatcataagcag
GUT1	F	atggcgcactgacgactcta
	R	tagccattggaccacagcac
GPP1	F	catcgaagttccaggtgctg
	R	tgtcacgggtaccagaggtg
GPP2	F	cattgaagtcccaggtgcag
	R	atcacgggtaccggaagttg
GPT2	F	accacccggaaatcatcaag
	R	tccgggatttccttgatttg
SCT1	F	tatggctcttggttgcatgg
	R	atggggtcaccgaattcaac
PHM8	F	ttgcaaagtgggttgtcgag
	R	atggataacatggcccatcc
GPM2	F	gcaagcacaccatccaatgt
	R	gcgtcaatccaaccacagaa

ENO1	F	tgacgaaggtggtgttgctc
	R	cttaccgtcgtgaccagcag
CDC19	F	cgatttgccagctttgtctg
	R	aaaacatcgttggcggttct
PCK1	F	cagaatcaaagtccgcgttg
	R	ctgaccagcgttccagacag
PDA1	F	cttcattcaaacgccaacca
	R	tgtcctcaggggccttctta
PDB1	F	ccgctttgaagggtttgaag
	R	tttgcagcggaattgacaac
THI3	F	gcaagctctttggggatctg
	R	ttgcggggaatttatccttg
PDC1	F	catgatcagatggggcttga
	R	gagcctttggaccgtgaatc
PDC5	F	gatcagaaacgccaccttcc
	R	ctgggacagcaacaggtttg
PDC6	F	gcctcacgcagagtacaacg
	R	tggcgatcttgtgattttcg
LPD1	F	gccacgggctctgaagttac
	R	aacccatttccaatccgatg
LAT1	F	ggctaagagggtgccagatg
	R	gtggcaacagcgactgagac
ACS1	F	ttttatgttgcgccaactgc
	R	acccaagcaacgcaaagatt
ACS2	F	ccttgggtaccgcctcaata
	R	tgggtagccttgtgacgttg
HFD1	F	gcgcacaaaaggaaaagctc
	R	ggcgtaacccccacttgtta
ADH1	F	cgttaagggctggaagatcg
	R	ccagacaagtcagcgtgagg
ADH2	F	cgttaagggctggaagatcg
	R	ccagacaagtcagcgtgagg
ADH3	F	aagccgccaaaattcaacag
	R	accagagatggcaacccagt
ADH4	F	gctgttgctgtcaacgatcc
	R	gaggcggtggaaacataagc
ADH5	F	tcgatggtggtaatgccaag
	R	actccatgagaaccgccatt

ADH6	F	tccatcacatttggctgctc
	R	tttttacctggaccgcaacc
ADH7	F	ggcgatcatgacgttgatgt
	R	tgggactggaccccaattac
SFA1	F	aatgactgatgggggtctgg
	R	ggcagccacaccaatgataa
CIT1	F	acccatggccaaatgttgat
	R	ggagcaccaacagccctatc
CIT2	F	attcggatcacgaaggtggt
	R	caaacctgatgcaagggaca
CIT3	F	tgcgttgagtgacccgtatc
	R	cttgtgctgctaacccatgc
ACO1	F	tgccatcaagagacccattg
	R	tccagcgtttccacattctg
IDP1	F	atgtgccaccatcactcctg
	R	cgccgagaatgtttctgatg
IDP2	F	tcggccaaatattggaaagc
	R	tagattccaccgcgtcaatg
IDP3	F	ataggccgtcatgcttttgg
	R	gcgatcccaccacttttagg
IDH1	F	atccgtccctggtgtagtgg
	R	ggcgaagtcaaaggcaaatc
IDH2	F	aaggtggtcaccaacccatc
	R	taaagaaccggcgctcaaac
KGD1	F	acccattcccatttgctcag
	R	tctgtgtatgcccacgaacc
LPD1	F	gccacgggctctgaagttac
	R	aacccatttccaatccgatg
KGD2	F	tattccagccgtcaatggtg
	R	ttacgaacgacgggggtaac
LSC1	F	atgggtggtgatgcttttcc
	R	ttcgatttcggccttaccac
LSC2	F	ggatttccagcgttcactcc
	R	cctctacctccggtcaatgc
SDH1	F	attcccacgaagtggaatgg
	R	ttggcaccatggacagaaac
SDH2	F	ggaaggccttttgtttggtg
	R	tagcacttggctcgtctgga

SDH3	F	ctccgccttttcaagagcag
	R	ccgctttggtgttcatttca
SDH4	F	caggtggtgttaggggcact
	R	acgacggacaaggcaaagat
SDH9	F	gggtctggctgcctaatcac
	R	ttccatggtgattgctctgg
SHH3	F	ccccgagcatctgtattcgt
	R	cggccattgtggaattcttt
SHH4	F	aaagcagagctcagggttcg
	R	accaagggaactgcgatcag
FUM1	F	aagaatgccattgcctttgg
	R	ggcctgttgaatagccttgg
MDH1	F	tccgtggtcaaggggtttac
	R	atggcgaacaagtcatcacg
MDH2	F	acatggaacgcaaccaaatg
	R	tcgatgcaacgaattccaag
MDH3	F	tggaaagttcgcaccaaatg
	R	ctgcacgtaccaggtcaagg
PCK1	F	cagaatcaaagtccgcgttg
	R	ctgaccagcgttccagacag
